# Retinoic acid and FGF10 promote the differentiation of pluripotent stem cells into salivary gland placodes

**DOI:** 10.1186/s13287-022-03033-5

**Published:** 2022-07-28

**Authors:** Siqi Zhang, Yi Sui, Shuang Yan, Yifei Zhang, Chong Ding, Xiaodong Su, Jingwei Xiong, Shicheng Wei

**Affiliations:** 1 Central Laboratory, and Department of Oral and Maxillofacial Surgery School and Hospital of Stomatology, Peking University, Beijing, 100081 People’s Republic of China; 2 Institute of Molecular Medicine, Peking University, Beijing, 100871 People’s Republic of China; 3 Laboratory of Biomaterials and Regenerative Medicine, Academy for Advanced Interdisciplinary Studies, Peking University, Beijing, 100871 People’s Republic of China; 4 Biomedical Pioneering Innovation Center (BIOPIC) State Key Laboratory of Protein and Plant Gene Research, School of Life Sciences, Peking University, Beijing, 100871 People’s Republic of China

**Keywords:** Pluripotent stem cells, Three-dimensional cultures, Salivary glands, Organogenesis, Modeling

## Abstract

**Background:**

Salivary glands produce saliva that play essential roles in digestion and oral health. Derivation of salivary gland organoids from pluripotent stem cells (PSCs) provides a powerful platform to model the organogenesis processes during development. A few studies attempted to differentiate PSCs into salivary gland organoids. However, none of them could recapitulate the morphogenesis of the embryonic salivary glands, and most of the protocols involved complicated manufacturing processes.

**Methods:**

To generate PSC-derived salivary gland placodes, the mouse embryonic stem cells were first differentiated into oral ectoderm by treatment with BMP4 on day 3. Retinoic acid and bFGF were then applied to the cultures from day 4 to day 6, followed by a 4-day treatment of FGF10. The PSC-derived salivary gland placodes on day 10 were transplanted to kidney capsules to determine the regenerative potential. Quantitative reverse transcriptase–polymerase chain reaction, immunofluorescence, and RNA-sequencing were performed to identify the PSC-derived SG placodes.

**Results:**

We showed that step-wise treatment of retinoic acid and FGF10 promoted the differentiation of PSCs into salivary gland placodes, which can recapitulate the early morphogenetic events of their fetal counterparts, including the thickening, invagination, and then formed initial buds. The PSC-derived salivary gland placodes also differentiated into developing duct structures and could develop to striated and excretory ducts when transplanted in vivo.

**Conclusions:**

The present study provided an easy and safe method to generate salivary gland placodes from PSCs, which offered possibilities for studying salivary gland development in vitro and developing new cell therapies.

**Supplementary Information:**

The online version contains supplementary material available at 10.1186/s13287-022-03033-5.

## Background

Pluripotent stem cells (PSCs) including embryonic stem cells (ESCs) and induced pluripotent stem cells (iPSCs) provide an ideal platform for investigating basic biological processes and developing cell-based therapies because of the remarkable ability to self-renew and differentiate into various cell lineages derived from all three germ layers in vitro [1–4]. The emergence of three-dimensional (3D) organoid culture conditions in vitro has raised the possibility that differentiation of PSCs can recapitulate the morphogenesis of the organs in many details, which display near-physiologic cellular composition and behaviors [5, 6]. Therefore, 3D organoid cultures hold advantages over two-dimensional (2D) differentiation systems. They allow the self-formation of specific structures and spatial control of cell patterning [7]. Previous studies have generated various organoids via the self-organization of PSCs and provided proof that organoids have unique opportunities to be considered as ideal tools in developmental biology and regenerative medicine [8–11].

Salivary glands (SGs) synthesize and secrete saliva through large quantities of epithelial acinar and complex connecting duct structures [12]. Saliva plays an essential role in digesting, swallowing, bacterial clearance, and maintaining overall oral health [13]. A few studies attempt to explore the protocol for inducing SG cells from PSCs. Most of them generated specific cell lineages by co-culture of PSCs with mature SG cells [14, 15]. Recently, researchers reported a more efficient protocol for directly differentiating PSCs into SG rudiments by overexpression of a critical signaling pathway and supplementing specific factors. After orthotopic transplantation, the SG rudiment was successfully developed to functional tissues, which provided the concept of SG organoids in next-generation organ replacement regenerative therapy [16]. However, these reported protocols have their shortcomings and limitations. None of them resemble the early morphogenesis events of developing SGs, which is essential for forming normal SGs with complex structures. Additionally, the manufacturing processes such as the outer epithelium resection and infection of adenoviruses required complicated procedures and had safety issues in clinical use.

During embryogenesis, the regulation of developmental processes is achieved by interactions of multiple signaling [17]. Therefore, signaling pathways underlying critical salivary gland developmental stages need to be identified to develop improved protocols for generating SG organoids. The development of submandibular glands (SMGs), one of the major SGs, begins with the thickening of the oral ectoderm (OE) and invagination of the earliest SG epithelium at embryonic day 11.5 (E11.5) in mice. Consequently, followed by complex biochemical and mechanical regulation, the invaginating epithelium continues to form initial buds extending into the neighboring condensed mesenchyme at E12.5 [18–20]. Multiple signaling pathways participate in SMG initiation and morphogenesis by mediating epithelial Sox9 expression [21]. Previous studies have reported that retinoic acid (RA) signaling regulates the earliest stage of epithelial invagination by promoting the expression of Sox9. In addition, the FGF10 remains Sox9 expression and plays an essential role in epithelial progenitor proliferation [22, 23]. These could be valuable clues to induce SG cells from PSCs.

Here, we reported an easy and safe protocol to generation SG placodes from mouse ESCs (mESCs) by treatment with RA and FGF10 directly. The PSC-derived SG placodes expressed specific genes known to be enriched in embryonic mouse salivary glands and formed initial buds and ducts. Furthermore, the differentiation process of initial buds recapitulated the early morphogenesis events, including thickening and invagination. In addition, the SG placodes showed luminal Krt19 positive duct cells and could develop to striated and excretory ducts in vivo, exhibiting the characteristics of mature SGs.

## Methods

### Maintenance of mESCs

Two mESCs cell lines, 129w2 and F15, were kindly provided by the Tang Fuchou laboratory at Peking University and maintained in serum-free and feeder-free culture conditions. The maintenance medium of mESCs (129w2) consisted of 1:1 mixture of KO-DMEM and Neurobasal medium supplemented with 0.5% N2 supplement, 1% B27 supplement, 50 μg/ml BSA, 2 mM glutamine, 0.1 mM β-mercaptoethanol, 1 mM penicillin/streptomycin, 0.1 mM NEAA, 3 μM CHIR99021, and 1 μM PD325901. Cells were cultured in six-well plates coated with 0.1% gelatin. The fresh medium was changed daily, and cells were passaged every three days.

### Differentiation of SG placodes from mESCs

The basic differentiation medium was as follows: G-MEM supplemented with 1% glutamine, 0.1 mM NEAA, 1 mM penicillin/streptomycin, 1% sodium pyruvate, 3% KO serum replacement, and 0.1 mM β-mercaptoethanol. When 60–70% confluence was reached, mESCs was detached into single cell and resuspended in differentiation medium. Cells were seeded in 96-well U-bottom ultra-low attachment plates at a density of 3,000 cells per well. The day on which seeding cells was defined is day 0. On day 1, half of the medium was changed and after which the medium must contain 2% growth factor reduced Matrigel. 2 ng/ml BMP4 was added to the cultures to stimulate the differentiation of OE on day 3. On day 4, the aggregates were treated with 25 ng/ml bFGF and 1 μM RA to induce the early salivary gland epithelium. On day 6, healthy aggregates were collected and transferred to 24-well ultra-low attachment plates with fresh medium containing 200 ng/ml FGF10, and the medium was changed every two days.

### RNA interference to knock down the expression of Sox9

The suppression of Sox9 was performed with short interfering RNA (siRNA), which was designed and synthesized by Sangon. FAM-labeled siRNA served as a positive control to detect whether the siRNA was transfected into cells. NC-siRNA was negative control lacking complementary RNA sequence. Lyophilized siRNA powder was resuspended in RNase-free water to a stock concentration of 20 μM. The PSC-derived SG placodes were transfected with 500 nM of FAM-siRNA, NA-siRNA, and three Sox9-siRNA using Lipofectamine3000 on day 6. To extend the duration of gene knockdown, the siRNAs were re-transfected on day 8.

### RNA sequencing and analysis

For RNA sequencing (RNA-seq), total RNA was extracted from 129w2 mESC-derived SG placodes on day 10 of differentiation using Rneasy mini kit (Qiagen), following manufacturer’s recommendations. RNA concentration and quality were measured using NanoDrop 2000 (Thermo Fisher Scientific, Wilmington, DE) and the Agilent Bioanalyzer 2100 system (Agilent Technologies, CA, USA). Sequencing libraries were prepared using NEBNext Ultra™ RNA Library Prep Kit for Illumina (NEB, USA). RNA sequencing was performed on Illumina Novaseq 6000 platform, and paired-end reads were generated. Hisat2 tools soft were used to map with the mouse reference genome. Quantification of gene expression levels was measured by fragments per kilobase of transcript per million mapped reads (FPKM).

### Kidney subcapsular transplantation

Nude mice are used in transplantation assay and used at 6–7 weeks of age. At least 10 PSC-derived SG placodes (day10) were collected and directly transplanted under the kidney capsule of nude mice. The kidney was harvested and the outgrowths were excised after one month of transplantation. The outgrowths were fixed and embedded in optical coherence tomography.

### Immunohistochemistry

The samples were harvested, fixed in 4% paraformaldehyde (PFA), and equilibrated in 30% sucrose overnight at 4 °C. Samples were embedded in optical coherence tomography and cryo-sectioned at a thickness of 5–10 μm. Sections were then treated with 0.1% Triton X-100 at room temperature for 10 min and blocked in 3% BSA for 2 h. Primary antibodies diluted in blocking solution were applied to the sections overnight at 4 °C. After washing 3 times with PBS, sections were incubated with secondary antibodies for 1 h at room temperature. At last, Nuclei were stained with DAPI and observed using a NIKON A1R-si confocal microscope. Hematoxylin and eosin staining was performed following a standard protocol. For alcian blue-periodic acid Schiff (AB-PAS) staining, the sections were incubated in alcian blue for 15 min and oxidized for 5 min using periodic acid. Followed by staining with Schiff reagent for 15 min, the nuclei were stained with hematoxylin and then captured using Olympus inverted microscope.

### RNA isolation and qPCR

Total RNA was isolated using RNeasy mini kit (Qiagen). Reverse transcription was carried out following the RevertAid First Strand complementary DNA Synthesis Kit (Thermo Fisher). Gene expression levels were determined by quantitative real-time polymerase chain reaction (qPCR). qPCR was performed using SybrGreen master mix (Roche) as follows: predenaturation at 95 °C for 10 min, followed by 40 cycles of 95 °C for 5 s and 60 °C for 30 s.

### Statistical analysis

All quantitative values are expressed as mean ± standard deviation (SD) of three or more independent experiments. Two-tailed, unpaired Student’s t-test was used to determine statistical significance. Multiple group comparisons were performed using one-way analysis of variance (ANOVA) followed by Tukey's multiple comparison test. Differences were considered significant at a *p* value of < 0.05, 0.01, and 0.001.Line art was created by OriginLab Origin 2019b and photographs were created by microscope manufacturer’s camera system. The boundaries of the epithelial layer of brightfield images were measured using ImageJ. Briefly, the aggregates are divided into the outer optically translucent epithelium and inner part, defined as the layers' boundaries. ImageJ can recognize the boundaries of two different adjacent parts, and we determined the accurate results by adjusting the threshold. Adobe Illustrator 2021 was used to create the combination art containing line drawing, extensive lettering, color diagrams.

## Results

### Sequential differentiation of mESCs to SG placodes by RA and FGF10

**Fig. 1:**
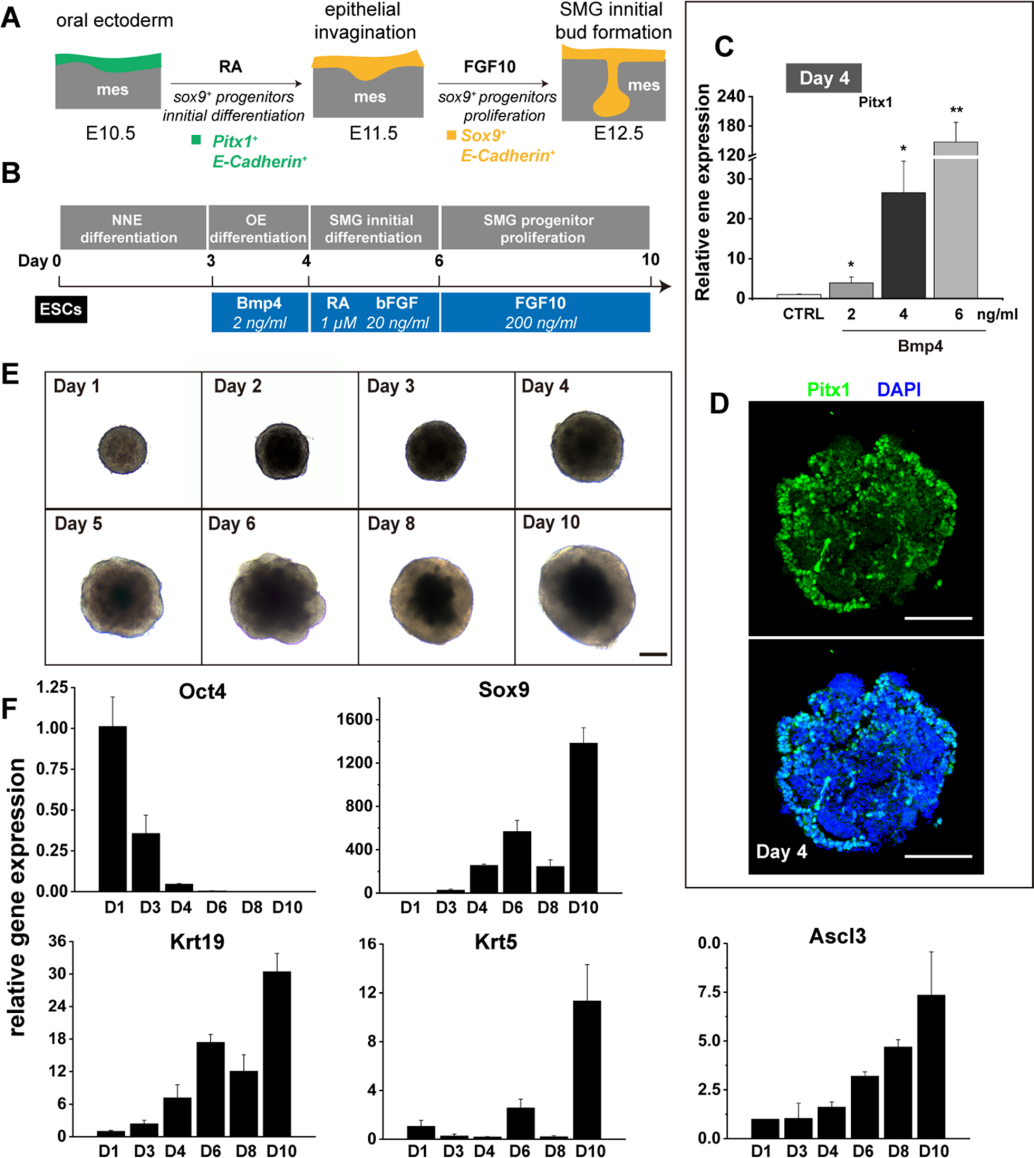
An overview of differentiation of mESCs to SG placodes. **A** Schematic representation of the early development of mouse SMG. **B** Culture protocol and schematic illustration of in vitro generation of SG placodes from embryonic stem cells (ESCs). NNE: non-neutral ectoderm; OE: oral ectoderm, SMG: submandibular glands. **C** BMP4 promoted the differentiation of oral ectoderm from mESCs. BMP4 increased the expression of Pitx1 in a dose-dependent manner. The mRNA expression was qualified by qPCR. The Ct values were compared to aggregates treatment without BMP4 (control: CTRL). **D** Immunofluorescence staining showed that Pitx1 positive oral ectoderm in the outer layer of aggregates at day 4. Scale bars, 100 μm. **E** Phase-contrast bright-light images represented the morphology of PSC-derived SG placodes during differentiation. Scale bars, 200 μm. **F** As differentiation proceeds, qPCR showed the increased expression of salivary gland progenitor markers. The Ct values were compared to aggregates on day 1. All qPCR results are presented as the fold change compared with the mean ± S.D. and were normalized to GAPDH in three independent experiments. *p < 0.05; **p < 0.01; ***p < 0.001 by unpaired, two-tailed Student’s t-test

SMG development begins with OE invaginating into the underlying mesenchyme at E11.5 (Fig. 1A). Therefore, mESCs were first differentiated into OE by treatment with BMP4 as described previously [16, 24]. The pluripotency of mESCs was maintained, indicated by the expression of Oct4 (Additional file 1: Fig. S1A-C). OE differentiation was started on day 3 (Fig. 1B), when the expression of non-neural ectoderm marker Dlx3 was upregulated, and before the neural ectoderm marker, Sox1 and Pax6 was downregulated (Additional file 1: Fig. S1D). Treatment with BMP4 at a final concentration of 2 ng/ml for 1 day was sufficient to promote the differentiation of the oral epithelium at the outer layer, which is indicated by the increased expression of Pitx1 (Fig. 1C, D). Of note is that the expression of T (mesoderm marker, also known as brachyury) was undetectable at all times in the cell aggregates (Additional file 1: Fig. S1D), which is inconsistent with previous studies [24]. Therefore, the combined treatment of SB431542 and BMP4 was unnecessary.

**Fig. 2:**
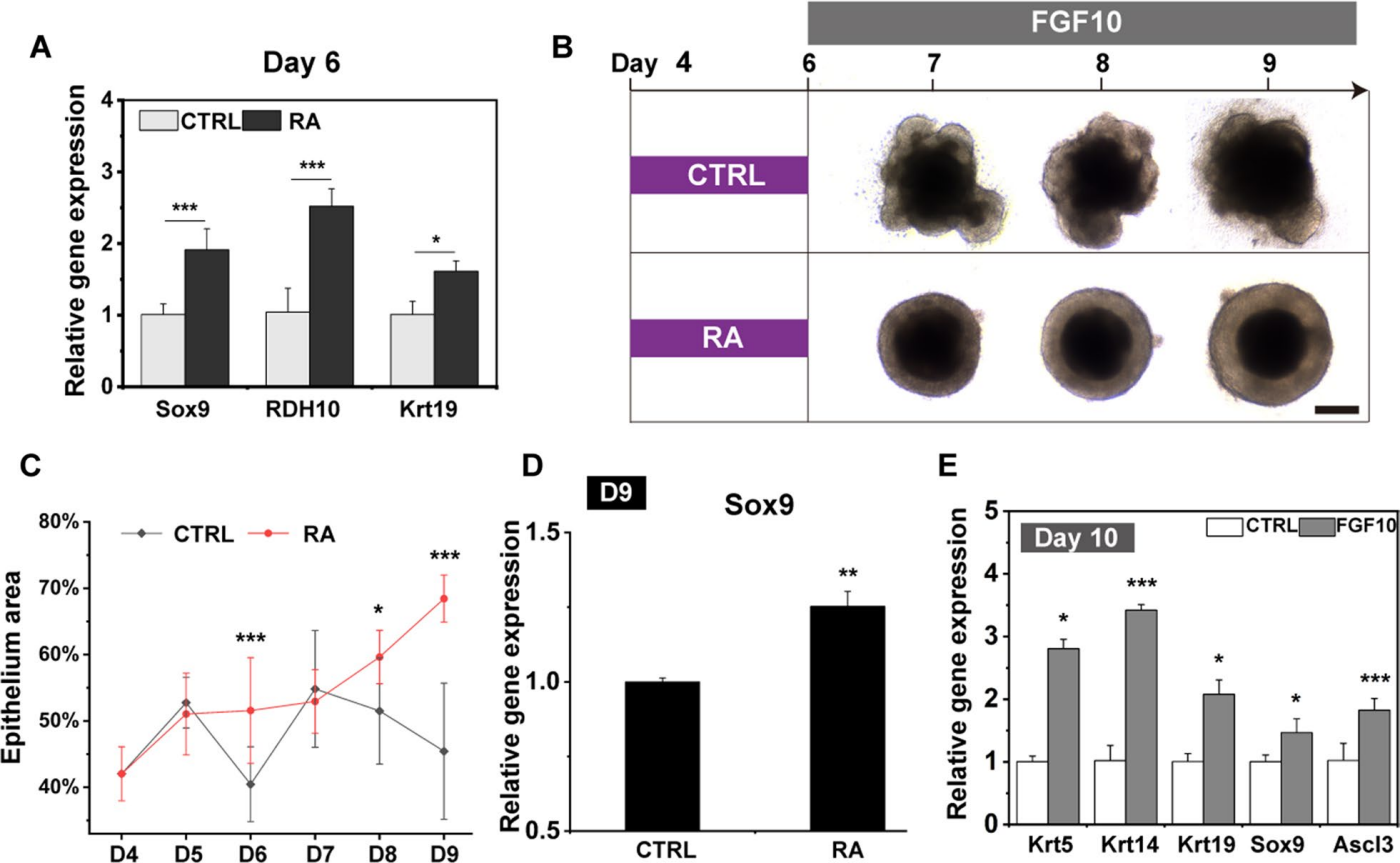
RA and FGF10 facilitated the differentiation of mESCs to SG placodes. **A** qPCR analysis revealed that salivary gland progenitor markers were upregulated after 2-days of RA treatment. The Ct values were compared to aggregates treatment without RA (control: CTRL). **B** The bright-light images of the aggregates in each culture condition (in the presence and absence of RA from day 4 to day 6) are shown. The aggregates cultured without RA failed to generate healthy SG placodes from day 7 to day 9. Scale bars: 200 μm. **C** The ratio of outer translucent salivary gland epithelium area became thicker after RA treatment, which became thinner in the control group (CTRL: aggregates cultured without RA). The area of cell death and outer epithelium were measured using ImageJ software. n > 5 independent experiments. **D** qPCR results showed that up-regulation of Sox9, but not Krt5 and Krt19, continued to day 9 in RA -stimulated aggregates. The Ct values were compared to aggregates treatment without RA (control: CTRL). **E** qPCR gene expression results after FGF10 treatment for 4 days, showed that salivary gland progenitor markers including Krt5, Krt14, Krt19, Sox9, and Ascl3 were upregulated. The Ct values were compared to aggregates treatment without FGF10 (control: CTRL). All qPCR results presented as the fold change compared with the mean ± S.D. and were normalized to GAPDH in three independent experiments. *p < 0.05; **p < 0.01; ***p < 0.001 by unpaired, two-tailed Student’s t-test

RA was then applied to the cultures from day 4 to day 6, followed by a 4-day treatment of FGF10 (Fig. 1B). During differentiation, the outer layer epithelium became thicker and expressed the increased levels of Sox9 and other salivary progenitor markers, including Krt5, Krt19, and Ascl3, but reduced levels of the pluripotency marker, Oct4 (Fig. 1E, F). Two-day treatment of RA increased the expression of Sox9 and Rdh10 coding gene (Fig. 2A), an enzyme of RA signaling [23]. These results indicated that RA signaling was activated. Expression of the SG duct progenitor gene Krt19 was also increased at this stage (Fig. 2A). Interestingly, we also found that the absence of RA from day 4 to day 6 failed to generate healthy aggregates even when cultured with FGF10 from day 6 to day 10 (Fig. 2B–D). These unsuitable aggregates showed more cell death and lacked smooth edges, most of which were dead on day 9 (Fig. 2B). In addition, the outer epithelium of these unsuitable aggregates was getting thinner during differentiation, which was different from aggregates treatment with RA from day 4 to day 6 (Fig. 2B, C). Upregulation of Sox9, but not Krt5 and Krt19, continued to day 9 in RA -stimulated aggregates (Fig. 2D), suggesting the essential role of RA in inducing the differentiation of salivary progenitor from OE and maintain the long-term culture. Furthermore, treatment with FGF10 produced a significant increase in expression of Sox9 and various salivary gland progenitor markers, including Krt5, Krt14, and Krt19 (Fig. 2E) [25, 26]. Notably, the expression of Ascl3, a gene expressed at the relatively late stage of salivary gland development [27, 28], was also increased (Fig. 2E). These data emphasize the importance of RA and FGF10 to induce the specification of SG epithelial progenitors from OE. In addition, we found that the aggregates became unhealthy on day 5 of differentiation (Additional file 1: Fig. S2A). Therefore, bFGF was applied to the culture to facilitate proliferation and reduce cell death in aggregates (Additional file 1: Fig. S2B). The results showed that the expression of Ki67 was increased after bFGF treatment but showed no effect on salivary differentiation (Additional file 1: Fig. S2C-D). Although the level of Sox9 was decreased after bFGF treatment (Additional file 1: Fig. S2D), the combination of bFGF and RA could promote SG initial differentiation through increased Sox9 expression (Fig. 2A). These results suggested that the combination of bFGF and RA promoted the initial differentiation of SGs and ensured the production of healthy SG aggregates in vitro.

### The PSCs-derived SG aggregates formed invaginating epithelium and developing duct-like structures

**Fig. 3:**
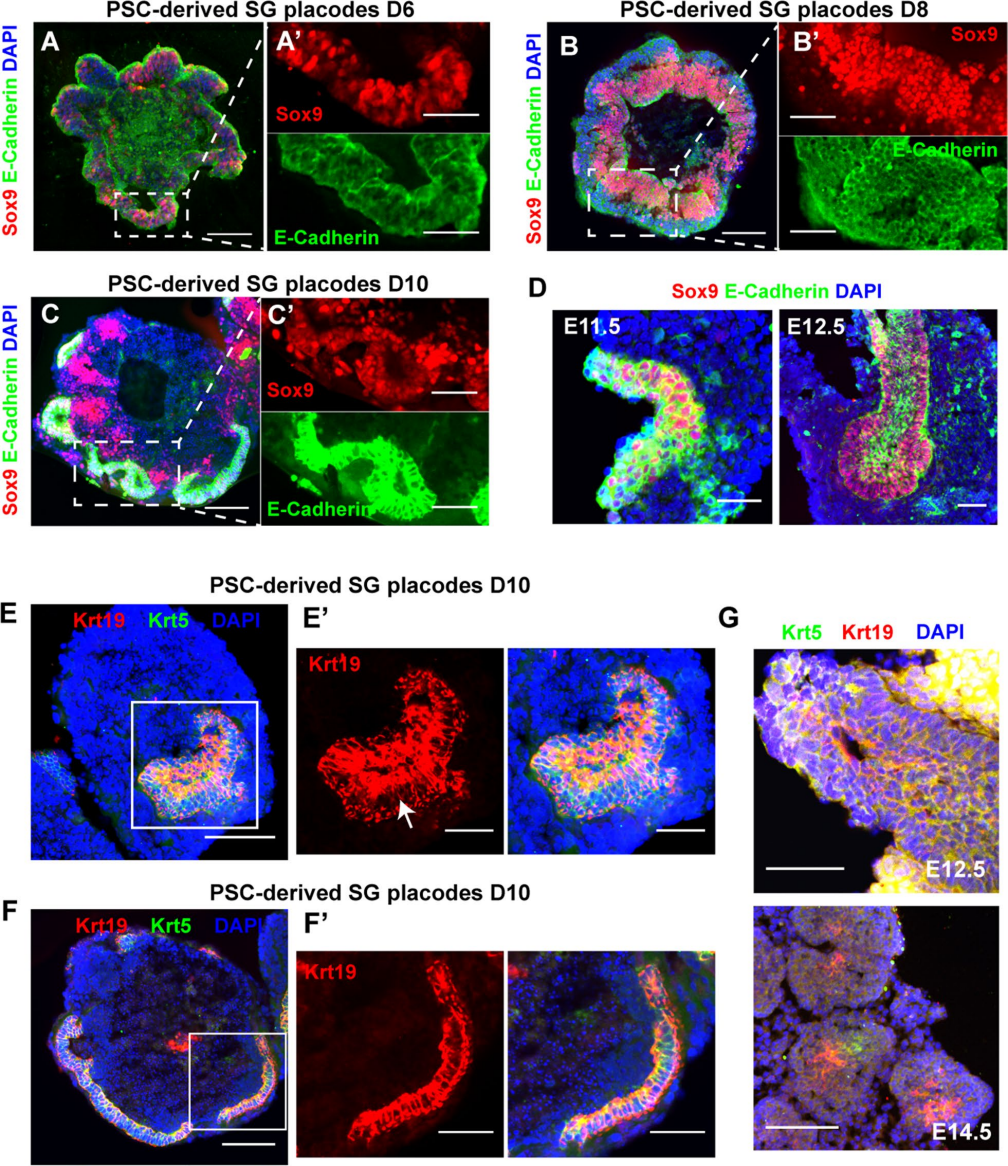
PSC-derived SG placodes formed invaginating epithelium and initial buds developing lumen-like structures. **A**–**C** PSC-derived SG placodes formed invaginating epithelium and initial bud-like structures from D6 to D10, which expressed a high level of Sox9 and E-Cadherin. Scale bars: 100 μm (**A**–**C**) and 50 μm (**A’**–**C’**, magnified images, marked by dotted boxes). **D** Immunofluorescence staining of E11.5 and E12.5 mouse submandibular glands. Green: E-Cadherin (Epithelium marker), red: sox9 (early-stage progenitor marker), blue: DAPI (nucleus). Scale bars: 50 μm. **E**–**F** The expression pattern of Krt5 and Krt19 in duct-like structures of SG placodes suggested polarized differentiation of lumen (a, marked by arrow), which was not observed in the invaginating epithelium (**F**) Scale bars: 100 μm (**E**, **F**) and 50 μm (**E’**, **F’**, magnified images, marked by boxes). **G** Immunofluorescence staining of E12.5 and E14.5 mouse submandibular glands. Green: Krt5 (salivary gland progenitor marker), red: Krt19 (lumen progenitor marker), blue: DAPI (nucleus). Scale bars: 50 μm (E12.5) and 100 μm (E14.5)

**Fig. 4:**
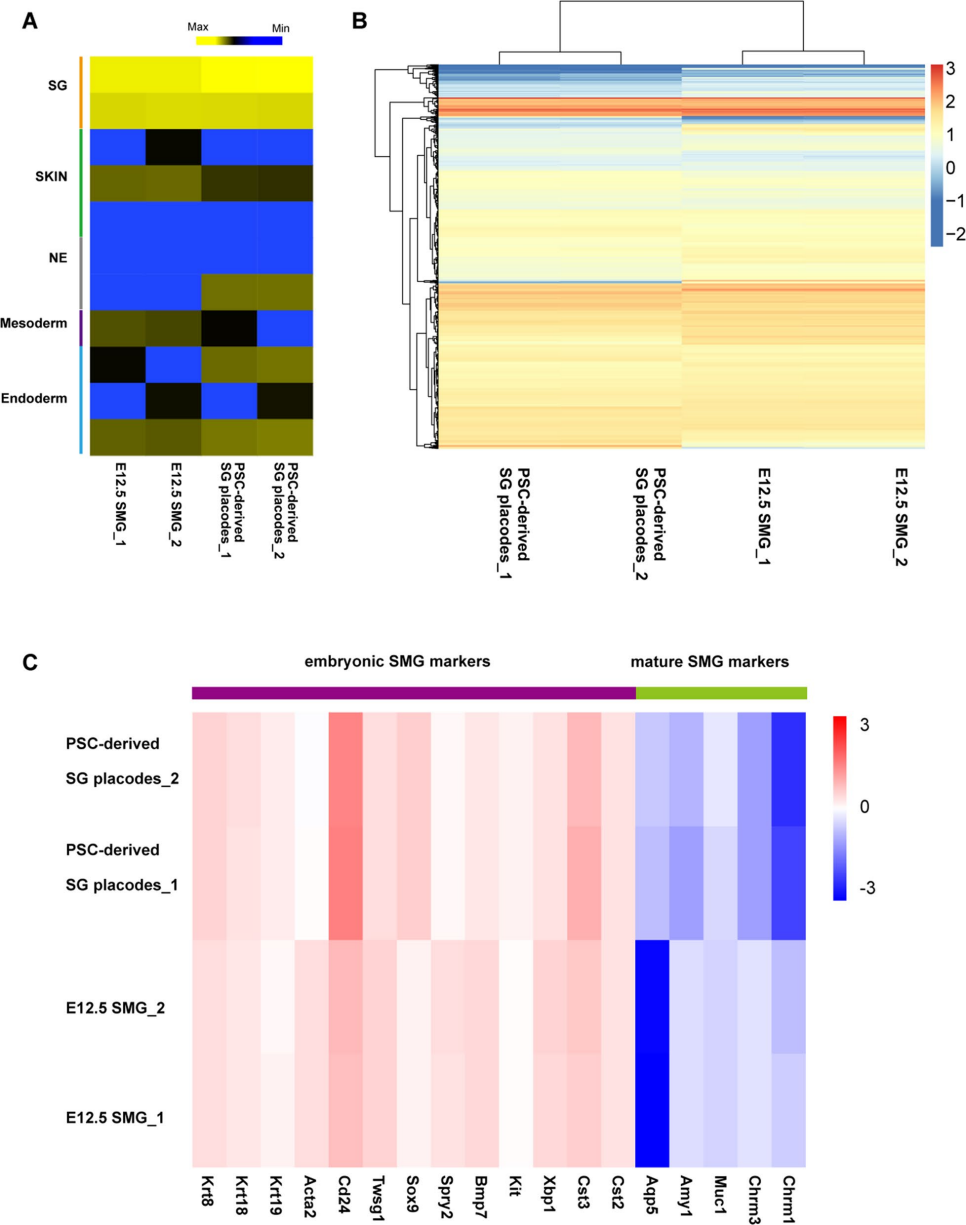
RNA-seq analysis revealed PSC-derived SG placodes resemble the transcriptome of E12.5 mouse SMGs. **A** The expression profile of key genes related to salivary gland (SG), skin, neural ectoderm (NE), mesoderm, and endoderm. **B** Hierarchical clustering analysis showed that the expression of genes related to development and morphogenesis was similar between PSC-derived SG placodes and E12.5 mouse SMGs. **C** A heatmap revealed expression profiles of key genes related to mouse embryonic and mature SMGs. *SMG* submandibular gland

We next evaluated the morphological changes and expression of salivary gland progenitor markers in protein levels. The Sox9 positive thinner epithelium was formed at the outer layer on day 6 (Fig. 3A). Following FGF10 treatment, the outer epithelium became optically translucent and gradually proliferated into thickening epithelium (Figs. 1E, 3B). Meanwhile, the Sox9 positive salivary early epithelium differentiated into a thickening placode on day 8 (Fig. 3B). Immunofluorescence analysis revealed that invaginating epithelium placode and initial buds were formed on the outer layers after two days. These structures co-expressed Sox9 and E-cadherin (Fig. 3C), normally enriched in the E11.5-E12.5 salivary gland early epithelium (Fig. 3D). During differentiation, the thickening placode, invaginating epithelium, and initial buds appeared in a timely manner similar to that described in embryonic SMGs [20]. Thus, these results suggested that the aggregates recapitulated the early morphogenetic process of embryonic salivary gland initial buds. Additionally, the expression of Krt5 and Krt19, key factors present in mouse embryonic SG duct epithelium, also increased during differentiation (Figs. 1F, 2E) [26]. Inspired by this result, we next examined whether the duct-like structures were formed. Immunofluorescence analysis showed that duct-like structures expressing Krt5 and Krt19 proteins were formed on day 10 (Fig. 3E). And Krt19 positive cells were condensed at the luminal side of the duct, which was not observed in the invaginating epithelium (Fig. 3E, F). These findings were consistent with developing SG ducts (Fig. 3G) [25]. Together these results demonstrated that the aggregates modeled the early morphogenesis events of developing SGs and the aggregates will be defined as PSC-derived SG placodes. In addition, we also found α-SMA positive cells at the outer layer of SG placodes, which are expressed in SGs through development and maturation (Additional file 1: Fig. S3A-B) [29]. RNA-seq was performed to further characterize the PSC-derived SG placodes. We first measured the expression level of the skin, neural ectoderm, mesoderm, and endoderm and ruled out the generation of other tissue derived cells (Fig. 4A). Through hierarchical clustering analysis, multiple development and morphogenesis-related genes expressed equally between PSC-derived SG placodes and E12.5 SMGs (Fig. 4B). A heatmap of genes associated with developing and mature SMGs showed that they had a similar gene expression profile to mouse E12.5 SMGs, suggesting that the SG placodes were in an undifferentiation state (Fig. 4C). Thus, RNA-seq revealed that PSC-derived SG placodes had a similar gene expression signature to mouse embryonic SMGs.

To demonstrate the importance of Sox9 during SG placodes differentiation, we used specific siRNAs to suppress the expression of Sox9 (Additional file 1: Fig. S4A). FAM-labeled siRNA was used as positive control and the positive signal was detected after 24 h (Additional file 1: Fig. S4B). All three Sox9-siRNAs inhibited the gene expression (Additional file 1: Fig. S4C). 4-day treatment of Sox9-siRNA had no impact on aggregate size but inhibited the differentiation of the salivary gland epithelium, indicated by decreasing thickness of epithelium at the outer layer (Additional file 1: Fig. S4D-F). Little invagination bud or duct-like structures were observed when Sox9 was knocked down (Additional file 1: Fig. S5A-B). The suppression of Sox9 also impacted duct differentiation and the expression of mature acinar markers α-SMA (Additional file 1: Fig. S5C).

**Fig. 5:**
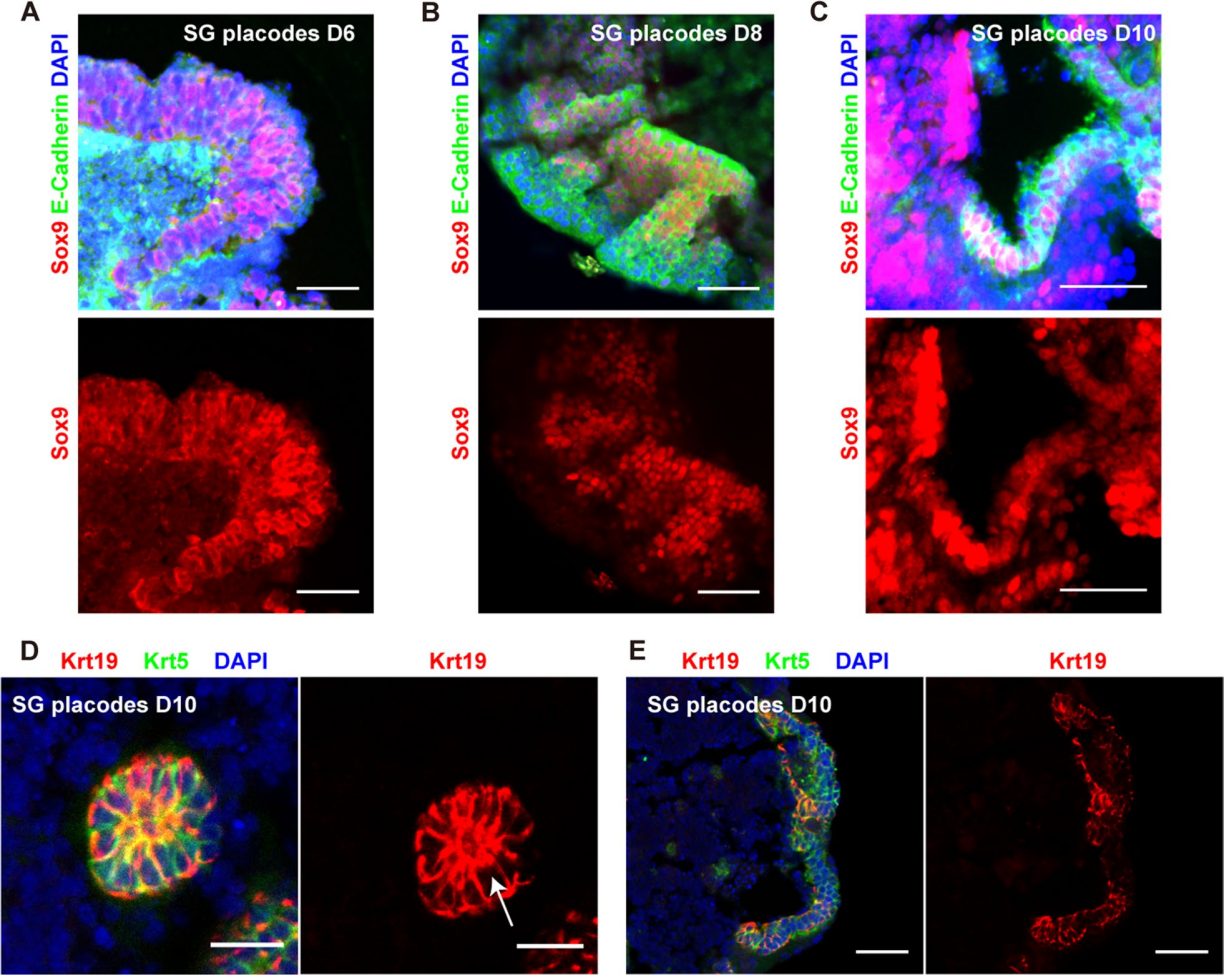
Generation of SG placodes from mESCs cell line F15. **A**–**C** Similar to 129w2 mESCs-derived SG placodes, the F15-derived SG placodes formed invaginating epithelium and initial bud-like structures from D6 to D10. Scale bars: 50 μm. **D**–**E** F15-derived SG placodes also showed polarized expression of Krt19 in developing ducts, but not in the invaginating epithelium. Scale bars: 25 μm (**D**) and 50 μm (**E**)

Another mESCs cell line, F15 mESCs, was induced following our protocol. Similarly, the F15 cell line successfully differentiated to thickening placode and Sox9 positive invaginating initial buds by RA and FGF10 treatment (Fig. 5A–C). Furthermore, F15-derived SG placodes also formed duct-like structures with polarized K19 positive cells (Fig. 5D, E), the same finding was observed in the 129w2 mESCs derived SG placodes. In addition, the expression of α-SMA was observed in F15 derived SG placodes (Additional file 1: Fig. S3C). Taken together, these data suggested that the protocol described above was robust to generate SG placodes from PSCs.

### PSC-derived SG placodes differentiated into mature salivary ducts in vivo

**Fig. 6:**
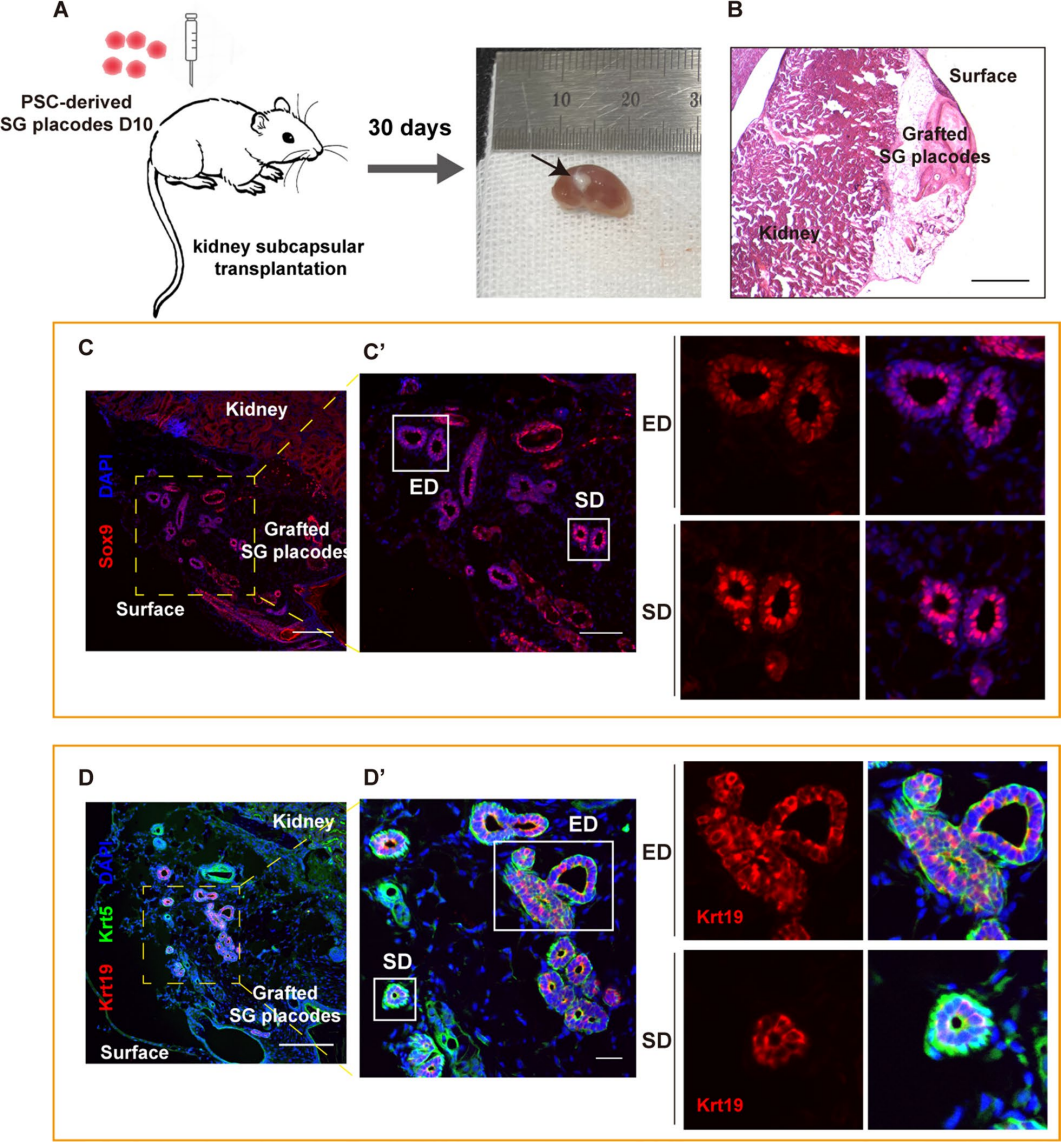
PSC-derived SG placodes differentiated into mature salivary ducts in vivo. **A** Schematic of transplantation procedures and photographs of mouse kidneys with grafted SG placodes after 30 days. (Black arrows marked grafted tissues). **B** Hematoxylin and eosin staining showed distinct outgrowths after transplantation for 30 days. Scale bars: 500 μm. **C**, **D** The excretory ducts (ED) and striated ducts (SD) were formed after transplantation of SG placodes under mouse kidney subcapsular. The mature ducts also expressed the high level of Krt19 (**C**) and Sox9 (**D**). Scale bars: 200 μm (**C**, **D**) and 100 μm (**C’** and **D’**, magnified images, marked by yellow dotted boxes)

To investigate whether the PSC-derived SG placodes could differentiate into mature salivary structures in vivo, the aggregates on day 10 were collected and directly transplanted under the kidney capsules of nude mice (Fig. 6A). The nascent tissues were observed 1 month after transplantation (Fig. 6A, B), suggesting the ability of SG placodes to proliferation and development in vivo. Histological analysis revealed a duct-like structure with contiguous lumens and elongated ducts developed in grafted SG placodes (Additional file 1: Fig. S6A), which was not observed in vitro (Figs. 3E, 5D). Of note is that the duct-like structures with a single layer of tall columnar and pseudostratified columnar epithelium were formed (Fig. 6C, D), which are the characteristics of salivary striated and excretory ducts, respectively (Additional file 1: Fig. S6B-D). In addition, the expression of salivary duct-specific markers, Krt19 and Sox9, was observed in both striated and excretory ducts (Fig. 6C, D), as seen in mouse SMG (Additional file 1: Fig. S6C-D). PSCs could differentiate to form teratoma in vivo. To test whether the mature duct-like structures were developed by undifferentiated mESCs, we measured the pluripotency of PSC-derived SG placodes on day 10. The expression of a pluripotent marker, Oct4, was not detected by qPCR and immunofluorescence staining (Fig. 1F and Additional file 1: Fig. S6E). In addition, we did not observe the signal of α-amylase, AQP5 and the secretion of mucin in duct structures within grafted SG placodes, which may be because of the absence of acinar-like structures (Additional file 1: Fig. S6F-G). These results demonstrated that the developing ducts in SG placodes are capable of differentiating into mature salivary ducts ectopically in vivo by mimicking the developmental process of mouse salivary ducts.

## Discussion

The present study induced the mESCs into SG placodes by step-wise treatment of RA and bFGF. The PSC-derived SG placodes recapitulated early-stage morphogenesis events, including thickening and invagination, and expressed similar markers of the SG progenitor. In addition, the SG placodes also differentiated into developing ducts and could develop to mature salivary duct-like structures with lumens and elongated ducts, as observed in mature salivary glands. Therefore, the PSC-derived SG placodes potentially serve as an ideal platform to establish developmental and disease models, which hold advantages over animal models, owing to being easier accessible and manipulatable.

The transcript factor Sox9 that is expressed in salivary gland epithelium at the initial stage and pre-bud stage modulates the formation and proliferation of distal epithelium [21]. Previous study reported that Sox9 was essential for the derivation of salivary gland rudiments from mESCs in vitro by infecting with a specific recombinant adenovirus [16, 21]. In the present study, the expression of Sox9 was promoted by direct treatment of RA. RA is synthesized from its precursor, retinol, by two reversible oxidation reactions [23]. The enzyme RDH10 and aldehyde dehydrogenase 1A play critical roles in RA signaling. Before and during salivary gland initiation, RA signaling is present in developing mandible tissues and thickening salivary gland placode. Previous studies revealed that RA signaling is essential to activate salivary gland initiation (E11.5), including thickening, invagination into the underlying mesenchyme, and the differentiation of progenitors. This process is transduced specifically through RA receptors α (RARα), and active RA signaling is required to activate Sox9 in the initiation of SMG development. We found that 2-day RA treatment promoted the expression level of Sox9 and other SG progenitors. Furthermore, the effect of RA treatment was presented even when RA was removed, demonstrating the critical role of RA in salivary initial differentiation. Previous studies have found that FGF10 regulates the proliferation of bud and duct progenitors and morphogenesis through their co-receptor FGFR2b during salivary gland development [22]. In addition, the expression of epithelial Sox9 is maintained by mesenchymal FGF10 during SMG development [21]. We showed that 4-day treatment of FGF10 successfully induced the thickening and invagination of SG epithelium placode and the formation of initial buds. And most SG progenitor markers were upregulated during the differentiation process. Therefore, our study demonstrated that Sox9 could be promoted by directly treating RA and FGF10 in PSC-derived SG placode cultures, as in mouse embryonic salivary glands. Additionally, bFGF treatment reduced cell death and promoted the proliferation of aggregates, which was essential for the long-term differentiation of PSCs in vitro. Taken together, this study provided proof that RA and FGF10 could induce the differentiation of salivary progenitor from PSCs.

Organoids are composed of multiple cell types and have complex structures, which allow them to mimic organ physiologic in many remarkable details. In this study, the PSCs-derived SG placodes could recapitulate the early morphogenetic events of mouse embryonic SMG, including thickening placode, invagination, and then form the Sox9 positive initial buds. Krt19 positive cells are the progeny of Krt5 progenitors, which modulate the differentiation of salivary ducts, and Krt19 positive duct cells polarize to form microlumens and condense at the lumen side in developing ducts [25, 26]. A similar structure was observed in the SG placodes, suggesting that our protocol could stimulate the early salivary duct development. RA signaling is also implicated in branching morphogenesis during SG development, which was not present in the SG placodes, suggesting that the interaction of multiple signaling is essential, and RA only was insufficient to induce the formation of branching structures.

To further determine the specification of SG placodes, we performed ectopic transplantation to determine whether they could develop to mature structures. We proved that the PSC-derived SG placodes could develop to striated and excretory ducts when transplanted under the kidney capsules, which proliferated to elongated ducts and formed lumens. Previous studies reported that Sox9 positive cells are located in intercalated ducts of mature salivary glands [21]. Here, we found Sox9 was also expressed in the striated and excretory duct of adult mouse SMG. And the duct cells in grafted SG placodes were Sox9 positive, as well as another duct-specific marker Krt19, consistent with our findings. The published study generated mESCs-derived salivary rudiment with acinar-like cells in vitro and showed that they can form mature tissues after orthotopic transplantation [16]. Our results from ectopic transplantation further demonstrated that RA and FGF10 are essential to inducing differentiation of developing salivary ducts in vitro. However, the acinar-like structure was absent and the grafted SG placodes lacked secretion of saliva, suggesting that an orthotopic microenvironment is necessary for salivary acinar development. Therefore, the in vivo transplantation experiment proved that we established an efficient protocol to induce SG placodes from PSCs. Furthermore, our results suggested that the PSC-derived SG placodes potentially serve as cell sources for repairing injured SGs. Further studies should perform orthotopic transplantation to determine the role of mESCs-derived SG placodes in regenerative therapies.

## Conclusions

In summary, we reported that direct treatment of two factors, RA and FGF10, facilitates the differentiation of SG placodes from mESCs, which is a more accessible and safer protocol. The PSC-derived SG placodes are capable of capitulating the early morphogenesis events of embryonic SGs, including thickening placode, invagination, and forming Sox9 positive initial buds. In addition, developing ducts with Krt19 expressed on the luminal side were found and could develop to mature salivary ducts with contiguous lumens in vivo. Therefore, the present study not only describes an easy and safe protocol to generate SG placodes from mESCs, but provides an opportunity to study the regulatory mechanisms underlying salivary gland organogenesis in vitro and develop new clinical therapy to achieve functional repair of SG hypofunction.

## Supplementary Information

**Additional file 1.** **Fig. S1**. The maintenance of mESCs and expression of three germ layer markers in aggregates. **Fig. S2**. The effect of bFGF during SG placodes differentiation. **Fig. S3**. The PSC-derived SG placodes expressed other salivary gland markers. **Fig. S4**. Suppression of Sox9 impaired the self-organization of SG placodes during morphogenesis. **Fig. S5**. The suppression of sox9 inhibited the expression of salivary markers. **Fig. S6**. SG placodes differentiated into mature salivary ducts in vivo.

## Data Availability

The data and materials used in this study are available from the authors on reasonable request. RNA-seq datasets of mouse E12.5 SMGs and PSC-derived SG placodes have been deposited in the GEO database under accession code GSE198489.

## Abbreviations

PSCs: Pluripotent stem cells
mESCs: Mouse embryonic stem cells
SGs: Salivary glands
SMGs: Submandibular glands
OE: Oral ectoderm
RA: Retinoic acid
