# Supplementary material for: Retinoic acid and FGF10 promote the differentiation of pluripotent stem cells into salivary gland placodes
Source: Stem Cell Res Ther. 2022 Jul 28;13:368. doi: 10.1186/s13287-022-03033-5 (PMC9330698; doi:10.1186/s13287-022-03033-5)
Supplement: Supplementary file 1 — Additional file 1. Fig. S1. The maintenance of mESCs and expression of three germ layer markers in aggregates. Fig. S2. The effect of bFGF during SG placodes differentiation. Fig. S3. The PSC-derived SG placodes expressed other salivary gland markers. Fig. S4. Suppression of Sox9 impaired the self-organization of SG placodes during morphogenesis. Fig. S5. The suppression of sox9 inhibited the expression of salivary markers. Fig. S6. SG placodes differentiated into mature salivary ducts in vivo. [file 13287_2022_3033_MOESM1_ESM.docx]

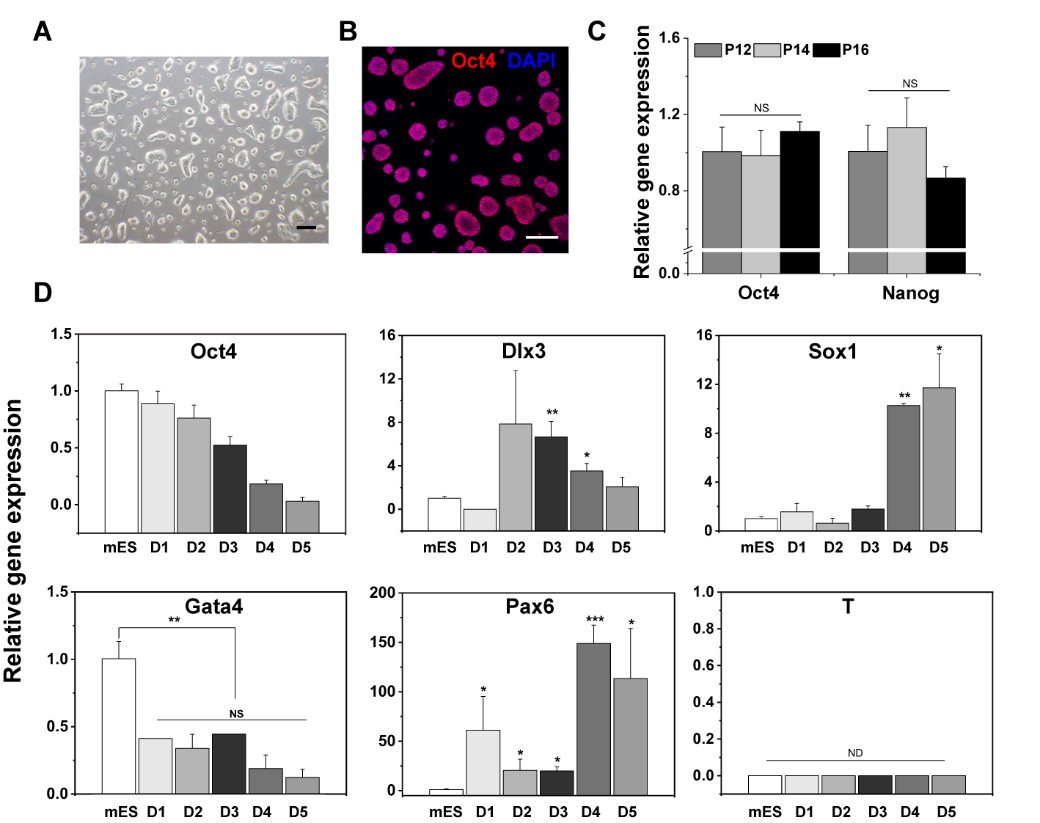


**Fig. S1 The maintenance of mESCs and expression of three germ layer markers in aggregates**

**(A)** Phase-contrast bright-light images represented the maintenance of mESCs. Scale bar: 200 μm.

**(B)** Immunofluorescence images showed Oct4 expression of mESCs. Scale bar: 200 μm.

**(C)** qPCR showed the expression of Oct4 had no significant (NS) changes during the maintenance of mESCs. The Ct values were compared to mESCs of P12. P: passage number.

**(D)** qPCR showed the expression of genes characterized in endoderm, ectoderm and mesoderm, which used to determine the beginning time of oral ectoderm differentiation. The Ct values were compared to aggregates at day 1. All qPCR results are presented as the fold change compared with the mean ± S.D. and were normalized to GAPDH in three independent experiments. * :p < 0.05, ** : p < 0.01 and *** : p < 0.001 by unpaired, two-tailed Student’s t-test. ND: nondetectable.


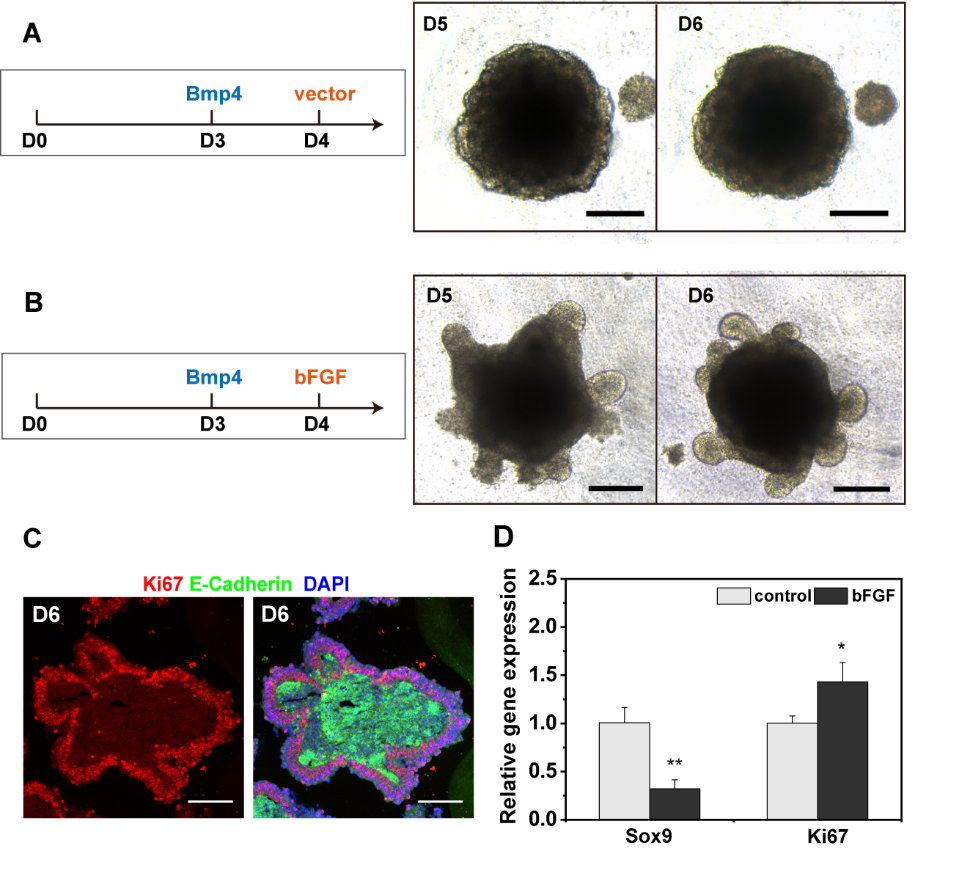


**Fig. S2 The effect of bFGF during SG placodes differentiation**

**(A-B)** The schematic illustration and phase-contrast bright-light images representing the morphogenesis of PSC-derived SG placodes in the presence (B) and absence (A) bFGF, showed that healthy aggregates were formed after bFGF treatment. Scale bars: 200 μm

**(C)** Immunofluorescence images showed the expression of Ki67 on the outer layer epithelium in aggregates cultured with bFGF. Scale bars: 100 μm.

**(D)** qPCR showed that the expression of Ki67 and Sox9 was increasing when aggregates were treated with bFGF. The Ct values were compared to aggregates without bFGF (control). All qPCR results are presented as the fold change compared with the mean ± S.D. and were normalized to GAPDH in three independent experiments. * :p < 0.05, by unpaired, two-tailed Student’s t-test.


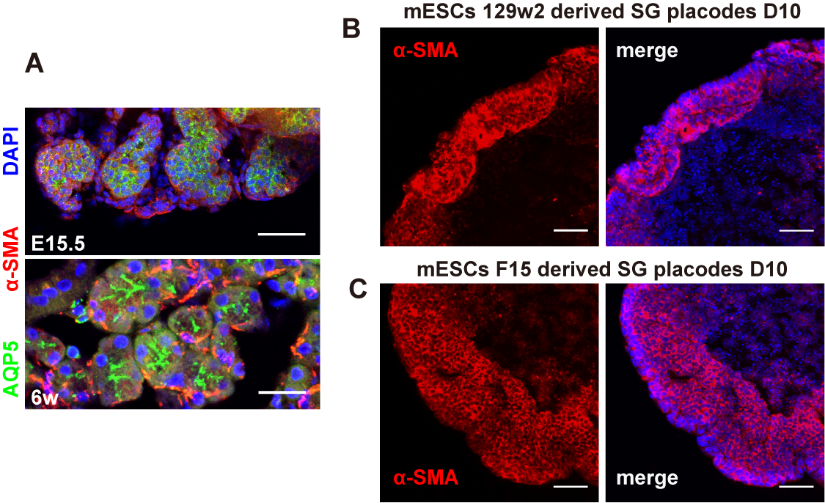


**Fig. S3 The PSC-derived SG placodes expressed other salivary gland markers**

**(A)** Immunofluorescence staining of E13.5 and 6 weeks mouse submandibular glands. Green: AQP5, red: α-SMA, blue: DAPI (nucleus). Scale bars: 100 μm.

**(B-C)** Immunofluorescence images showed the expression of α-SMA of two mESCs cell lines, 129w2 and F15 derived SG placodes at day 10. Scale bars: 50 μm.


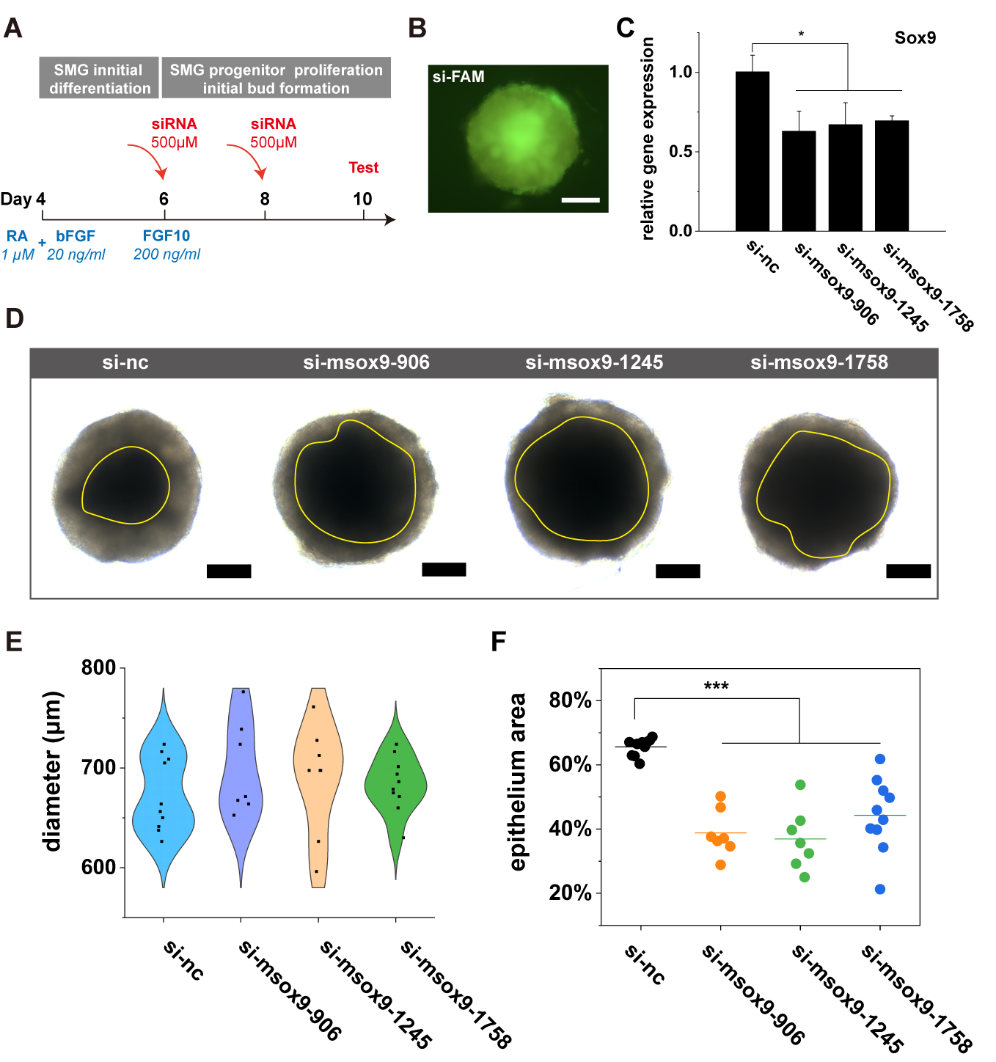


**Fig. S4 Suppression of Sox9 impaired the self-organization of SG placodes during morphogenesis**

**(A)** Schematic illustration of the Sox9 suppression experiment using siRNA. Negative control (NC) and Sox9-siRNA were applied to SG placode cultures from day 6 to day 10 in a final concentration of 500nM.

**(B)** Immunofluorescence images showed that FAM-label siRNA was successfully transfected into the PSC-derived SG placodes. Scale bar: 200 μm.

**(C)** qPCR showed the expression of Sox9 was down-regulated when cultured with each siRNA. The Ct values were compared to aggregates cultured with NC-siRNA (si-nc).

**(D)** Phase-contrast bright-light images represented the suppression of Sox9 impaired the thickening of the outer epithelium. Yellow circles marked the dividing line between outer epithelium and inner cell debris. Scale bars: 200 μm.

**(E)** Suppression of Sox9 showed no effect on the size of SG placodes treated with Sox9-siRNA. SG placodes treatment with NC-siRNA (si-nc) was included as control.

**(F)** The ratio of outer epithelium showed the suppression of Sox9 impaired the differentiation of out epithelium. The diameter and area of cell death (defined as: D) and whole epithelium (defined as: W) were measured using ImageJ software. The ratio of outer epithelium was measured as follows: (W-D)/W. n＞5 independent experiments; unpaired two-tailed t-test. *** :p < 0.001. ll qPCR results were presented as the fold change compared with the mean ± S.D. and were normalized to GAPDH in three experiments, * :p < 0.05 by unpaired and two-tailed t-test.


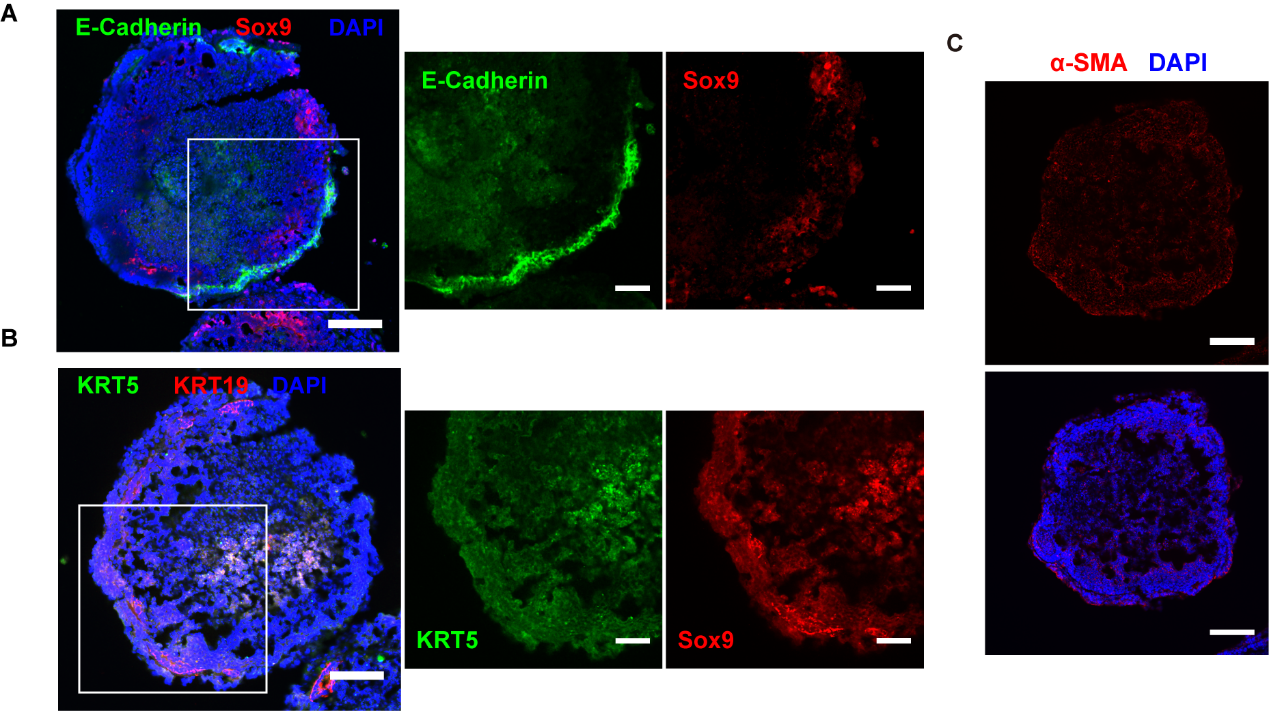


**Fig. S5 The suppression of sox9 inhibited the expression of salivary markers**

**(A-B)** Immunofluorescence staining of E-Cadherin and Sox9 (A), Krt5 and Krt19 (B). Scale bars: 50 μm.

**(C)** Immunofluorescence images showed the expression of AQP5 and α-SMA when Sox9 was knocked down. Scale bars: 100 μm.


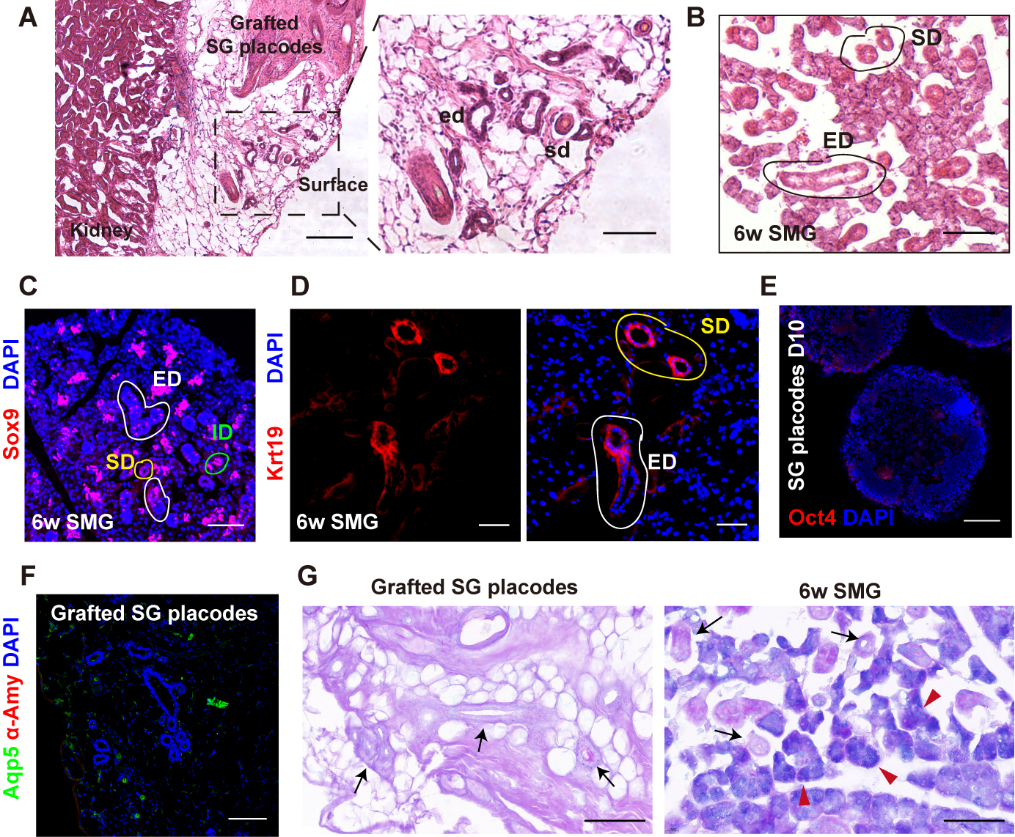


**Fig. S6** **SG placodes differentiated into mature salivary ducts in vivo**

**(A)** Hematoxylin and eosin staining showed the formation of mature ducts with contiguous lumens and elongated ducts after transplantation for 30 days. Scale bars:200 μm (a) and 100 μm (magnified images, marked by boxes).

**(B)** Hematoxylin and eosin staining showed the duct structures in adult (6 weeks) mouse submandibular glands. Scale bars: 100 μm.

**(C-D)** The excretory ducts (ed) and striated ducts (sd) of adult mouse (6w) submandibular glands express Sox9 (C) and Krt19 (D) and consist of contiguous lumens and elongated ducts. Scale bars:50 μm. ED: excretory ducts; SD: striated ducts; ID: intercalated ducts.

**(E)** Immunofluorescence images showed the SG placodes on day 10 did not express the pluripotent marker, Oct4. Scale bar: 100 μm.

**(F)** Immunofluorescence of Aqp5 and α-Amylase in grafted SG placodes. Scale bar: 100μm.

**(G)** AB-PAS staining of grafted SG placodes and mouse mature SMGs showed negative signal of Mucins. Scale bars: 100μm.
